# Novel technique using surgical scrub sponges to protect the nose and face during prone ventilation for coronavirus disease 2019

**DOI:** 10.1017/S0022215120001590

**Published:** 2020-07-28

**Authors:** T J Stubington, M S Mansuri

**Affiliations:** Department of Otorhinolaryngology – Head and Neck Surgery, Royal Derby Hospital, UK

**Keywords:** Prone Position, Ventilation, Pressure

## Abstract

**Background:**

Coronavirus disease 2019 is an international pandemic. One of the cardinal features is acute respiratory distress syndrome, and proning has been identified as beneficial for a subset of patients. However, proning is associated with pressure-related side effects, including injury to the nose and face.

**Method:**

This paper describes a pressure-relieving technique using surgical scrub sponges. This technique was derived based on previous methods used in patients following rhinectomy.

**Conclusion:**

The increased use of prone ventilation has resulted in a number of referrals to the ENT team with concerns regarding nasal pressure damage. The described technique, which is straightforward and uses readily available materials, has proven effective in relieving pressure in a small number of patients.

## Introduction

Coronavirus disease 2019 (Covid-19) in particular affects the lungs, causing an acute respiratory distress syndrome type picture. This results in an atypical form of acute respiratory distress syndrome, whereby there is disproportionately poor oxygenation despite reasonably preserved lung compliance in the early stages.^1^ Experience from Italy and China suggests that nursing the patient in a prone position is potentially beneficial, and can improve outcomes when carried out in the early stages of the disease.^1,2^ This has resulted in its inclusion in several international guidelines and adoption around the world as a valid intervention for Covid-19 patients.^3^

Proning is not a new phenomenon and it has been used as a treatment option for acute respiratory distress syndrome for over 20 years. However, it is not without complications. Proning can result in the displacement of tubes and lines, and the exacerbation of existing traumas or dehiscence of surgical wounds. There are also reports of pressure necrosis secondary to prone positioning, particularly of the face and nose.^4^ A Cochrane review in 2015 concluded that prone ventilation was directly responsible for an increased risk of pressure sores.^5^ There is some suggestion that the pressure damage caused by proning occurs regardless of the adoption of preventative measures (such as foam supports and other measures to relieve pressure).^4^ Nevertheless, this pressure damage is often mild and self-resolving.^6^ Regular repositioning of the head may also reduce pressure damage accordingly.^7^

As larger numbers of patients are likely to be prone, and given that proning is directly linked to pressure damage to the face and nose, it would seem logical that this would represent an increase in referrals to ENT to assess this. Anecdotally, our department has received several calls regarding such issues, having never previously encountered this complication in routine practice. Although patients should be prone with the head turned to one side to avoid such pressure damage,^8^ given the highly unstable nature of Covid-19 patients, and in some cases limited cervical spine rotation, inevitably some patients will end up in positions where their nose is at risk. We present our approach to the management of these injuries, borrowing from theory and practice used to manage patients who underwent rhinological procedures.

## Materials and methods

Our approach focuses on the following points. The first point concerns easily accessible, readily available materials. Given the demand that healthcare services are currently under around the world, a complex and expensive solution requiring multiple bespoke components is undesirable. The second point relates to being respectful of surrounding anatomical structures. If the technique is not applied with consideration of the surrounding structures, there is the risk that a nasal injury will be avoided but at the expense of corneal or labial injuries. Third, the method should allow for easy monitoring. Proning presents a significant challenge for visualisation of the nose and eyes. Furthermore, bulky padding risks obscuring the affected area, preventing monitoring. Skin breakdown and infection are a real concern with pressure damage in this area. Finally, the technique should be easily adaptable. A bespoke solution for each patient may be effective, but it will be expensive, and will likely take time to produce or acquire.

Surgical sponge has already been described as an appropriate material to immobilise and protect delicate grafts.^9^ It is our practice to use surgical sponge in the early stages of recovery following rhinectomy. It is our opinion that surgical scrub sponges meet the criteria set out above, and they represent an effective method of pressure relief in patients being ventilated prone. First, they are an easily accessible and readily available material. These sponges are available in all operating theatres and are bought in bulk at relatively low cost. Second, the sponges can be used in a manner that is respectful of surrounding structures. The sponges can be adjusted and cut to avoid nearby structures. Third, the use of sponges allows for easy monitoring. The sponges can be moved and the nose viewed. Fourth, the sponges can be easily adapted. They can be cut to any size, and additional sponges can be employed to account for larger anatomy.

**Fig. 1.**
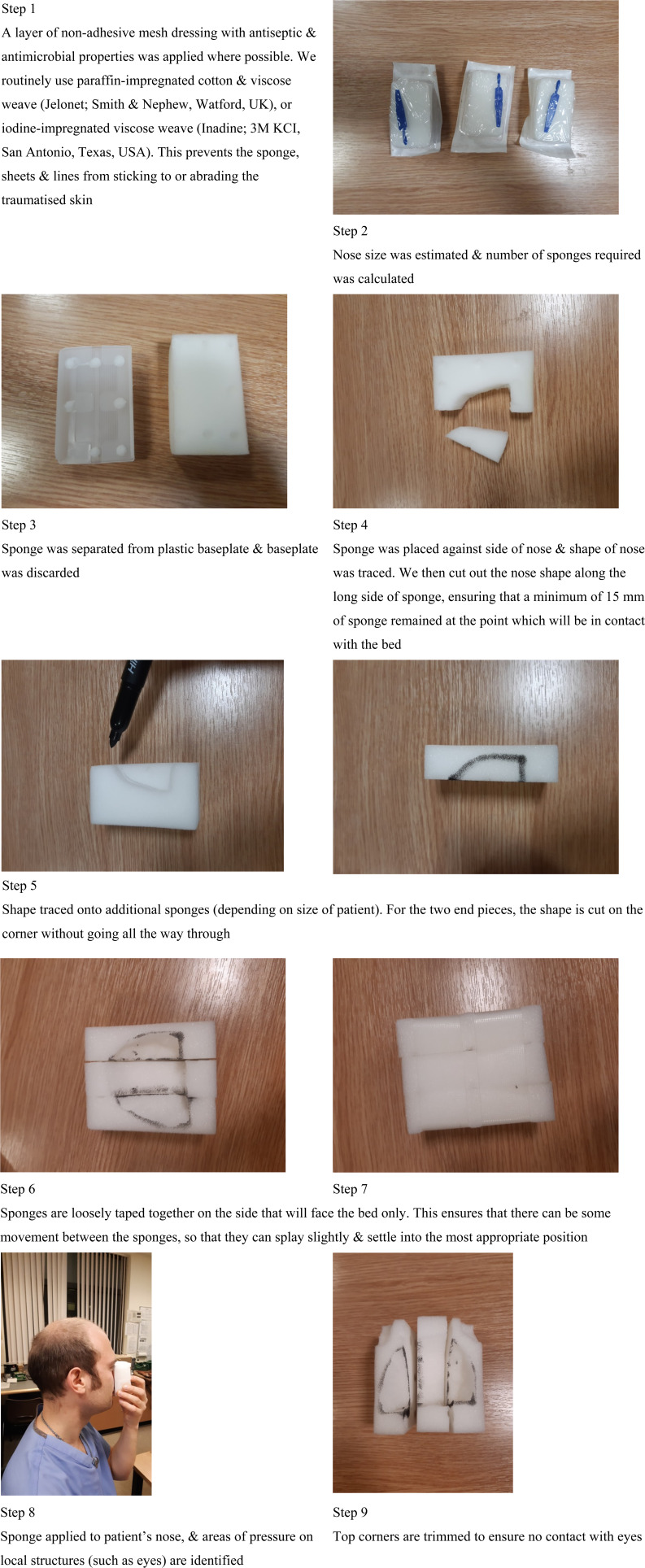
Step-by-step approach used to cut the surgical sponge to size and fit it to the patient's face.

The steps taken to measure and apply the sponge are detailed in Figure 1.

**Fig. 2.**
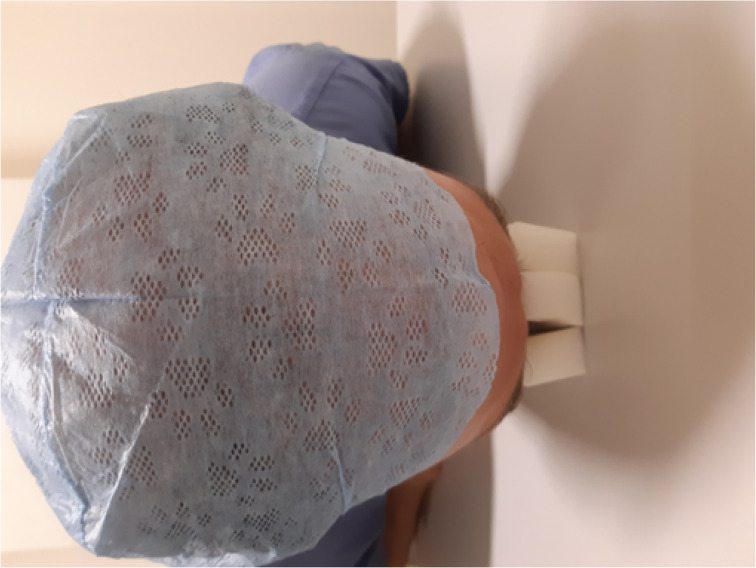
Top down view of the three sponges in situ; this demonstrates the slight splaying, which redistributes pressure.

**Fig. 3.**
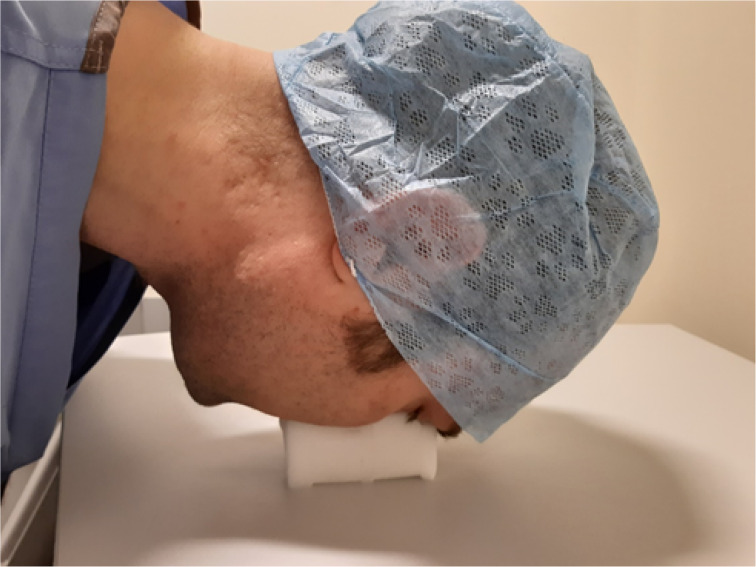
Lateral view of the sponge in situ.

The solution in situ can be seen in Figures 2 and 3. As shown in Figure 2, because the sponges are only secured together at one edge, they can splay slightly. This allows the pressure to be re-distributed, and stops the sponge bulging and encroaching on the eyes.

## Conclusion

We describe a simple technique used to relieve pressure from the nose and face in patients who are ventilated prone. The technique focuses on low cost, easily available materials, which allow easy monitoring of the area and that can easily be adapted to the individual patient. With the increasing use of prone ventilation to treat Covid-19 patients, it is our feeling that the described technique can reduce the incidence of proning-induced pressure injuries to the face and nose.

## References

[1] Gattinoni L, Coppola S, Cressoni M, Busana M, Rossi S, Chiumello D. COVID-19 does not lead to a “typical” acute respiratory distress syndrome. Am J Respir Crit Care Med 2020;201:1299–300 32228035 10.1164/rccm.202003-0817LE PMC7233352

[2] Meng L, Qiu H, Wan L, Ai Y, Xue Z, Guo Q Intubation and ventilation amid the COVID-19 outbreak: Wuhan's experience. Anesthesiology 2020;132:1317–32 32195705 10.1097/ALN.0000000000003296 PMC7155908

[3] Alhazzani W, Møller MH, Arabi YM, Loeb M, Gong MN, Fan E Surviving Sepsis Campaign: guidelines on the management of critically ill adults with coronavirus disease 2019 (COVID-19). Intensive Care Med 2020;46:854–87 32222812 10.1007/s00134-020-06022-5 PMC7101866

[4] Offner PJ, Haenel JB, Moore EE, Biffl WL, Franciose RJ, Burch JM. Complications of prone ventilation in patients with multisystem trauma with fulminant acute respiratory distress syndrome. J Trauma 2000;48:224–8 10697078 10.1097/00005373-200002000-00004

[5] Bloomfield R, Noble DW, Sudlow A. Prone position for acute respiratory failure in adults. Cochrane Database Syst Rev 2015;(11):CD008095 10.1002/14651858.CD008095.pub2 PMC6464920 26561745

[6] Mancebo J, Fernández R, Blanch L, Rialp G, Gordo F, Ferrer M A multicenter trial of prolonged prone ventilation in severe acute respiratory distress syndrome. Am J Respir Crit Care Med 2006;173:1233–9 16556697 10.1164/rccm.200503-353OC

[7] Messerole E, Peine P, Wittkopp S, Marini JJ, Albert RK. The pragmatics of prone positioning. Am J Respir Crit Care Med 2002;165:1359–63 12016096 10.1164/rccm.2107005

[8] Intensive Care Society. Prone Position Guidance in Adult Critical Care. In: https://ics.ac.uk/ICS/ICS/Pdfs/Prone_Position_Guidance_in_Adult_Critical_Care.aspx [3 May 2020]

[9] Yiacoumettis AM, Papadimitriou AM, Topkas AT, Kyrmizoglou PS. Letter: Polyurethane sponge from plastic scrubbing brush used as a pressure dressing over skin graft. Dermatol Surg 2007;33:1406–7 17958604 10.1111/j.1524-4725.2007.33304.x

